# Pathogens in space: Advancing understanding of pathogen dynamics and disease ecology through landscape genetics

**DOI:** 10.1111/eva.12678

**Published:** 2018-07-28

**Authors:** Christopher P. Kozakiewicz, Christopher P. Burridge, W. Chris Funk, Sue VandeWoude, Meggan E. Craft, Kevin R. Crooks, Holly B. Ernest, Nicholas M. Fountain‐Jones, Scott Carver

**Affiliations:** ^1^ School of Natural Sciences University of Tasmania Hobart Tasmania Australia; ^2^ Department of Biology Graduate Degree Program in Ecology Colorado State University Fort Collins Colorado; ^3^ Department of Microbiology, Immunology, and Pathology Colorado State University Fort Collins Colorado; ^4^ Department of Veterinary Population Medicine University of Minnesota St. Paul Minnesota; ^5^ Department of Fish, Wildlife, and Conservation Biology Colorado State University Fort Collins Colorado; ^6^ Wildlife Genomics and Disease Ecology Laboratory Department of Veterinary Sciences University of Wyoming Laramie Wyoming

**Keywords:** disease ecology, infectious disease, landscape epidemiology, landscape genetics, pathogen dynamics

## Abstract

Landscape genetics has provided many insights into how heterogeneous landscape features drive processes influencing spatial genetic variation in free‐living organisms. This rapidly developing field has focused heavily on vertebrates, and expansion of this scope to the study of infectious diseases holds great potential for landscape geneticists and disease ecologists alike. The potential application of landscape genetics to infectious agents has garnered attention at formative stages in the development of landscape genetics, but systematic examination is lacking. We comprehensively review how landscape genetics is being used to better understand pathogen dynamics. We characterize the field and evaluate the types of questions addressed, approaches used and systems studied. We also review the now established landscape genetic methods and their realized and potential applications to disease ecology. Lastly, we identify emerging frontiers in the landscape genetic study of infectious agents, including recent phylogeographic approaches and frameworks for studying complex multihost and host‐vector systems. Our review emphasizes the expanding utility of landscape genetic methods available for elucidating key pathogen dynamics (particularly transmission and spread) and also how landscape genetic studies of pathogens can provide insight into host population dynamics. Through this review, we convey how increasing awareness of the complementarity of landscape genetics and disease ecology among practitioners of each field promises to drive important cross‐disciplinary advances.

## 1 INTRODUCTION

The field of landscape genetics seeks to identify relationships between heterogeneous landscape features and genetic variation in free‐living organisms and has become a popular method for investigating drivers of processes such as gene flow, genetic drift and selection. (Manel & Holderegger, 2013; Manel, Schwartz, Luikart, & Taberlet, 2003). Landscape genetics has grown substantially since its formal inception in 2003, facilitated by technological advances that have increased the availability of molecular and landscape data in conjunction with more powerful computational and analytical approaches. Landscape genetics is fuelled by a steady stream of new ideas and methodologies, which, while exciting, can contribute to a lack of consensus or consistency in some key aspects. These aspects include the formulation of research questions, sampling strategies, analytical methods (Balkenhol, Waits, & Dezzani, 2009; Richardson, Brady, Wang, & Spear, 2016; Wagner & Fortin, 2013) and even the identity of the field itself (Dyer, 2015; Storfer et al., 2007). In fact, landscape genetics has yet to develop its own comprehensive, unifying theory for linking spatial and temporal landscape heterogeneity to genetic variation (Balkenhol, Cushman, Waits, & Storfer, 2016). While these issues are expected to be remedied as the field matures, many suggestions have been made to facilitate this progress. These have included calls for an increase in cross‐disciplinary collaboration (Balkenhol, Gugerli et al., 2009) and an expansion of the scope of landscape genetic research beyond its current emphasis on vertebrates (Balkenhol, Cushman, Waits et al., 2016; Dyer, 2015) and, particularly, mammals (Kozakiewicz, Carver, & Burridge, 2018).

One logical avenue for cross‐disciplinary expansion of landscape genetics is in disease ecology (Biek & Real, 2010). Elucidating the specific influences of landscape features on pathogen transmission can provide key insights into the processes that affect disease risk and incidence. However, accomplishing this has been a challenge for disease ecologists (Ostfeld, Glass, & Keesing, 2005). Indeed, the field of spatial epidemiology has only recently begun to emphasize the use of explicit landscape approaches in studies of spatial heterogeneity in infectious disease (i.e., “landscape epidemiology”; Ostfeld et al., 2005; Meentemeyer, Haas, & Václavík, 2012). A major challenge for the study of landscape epidemiology, a field which does not traditionally implement genetic approaches, is that it is typically dependent on the ability to identify the location and timing of transmission events such that they can be compared to landscape features of interest. Transmission events are essentially impossible to observe, so disease ecologists often assume that contacts between infected and susceptible individuals are a reasonable proxy for transmission. Such contacts generally must be inferred indirectly using methods such as proximity collars, mark‐recapture or telemetry, often using spatial overlap as a proxy for contact (Craft & Caillaud, 2011). These methods are logistically challenging to employ, and whether an inferred contact resulted in transmission is uncertain (Craft, 2015). Further, much landscape epidemiological research uses infection or exposure data to indicate past transmission, but these methods provide static snapshots of pathogen prevalence and may be inappropriate for inferring how transmission or spread has occurred (or is occurring) over time (Meentemeyer et al., 2012).

The spatial distribution and movement of hosts are major factors affecting the likelihood, timing and spatial patterns of pathogen transmission and spread (Dougherty, Seidel, Carlson, Spiegel, & Getz, 2018). Landscape genetics can identify landscape factors that are important drivers of host population structure. These landscape factors can determine the spatial configuration of a population, its density, its connectivity with other populations, its demographic structure and its genetic health—all of which have implications for the dynamics of microorganisms infecting the host species (Ellis, Václavík, & Meentemeyer, 2010; Prentice, Marion, White, Davidson, & Hutchings, 2014; Spielman, Brook, Briscoe, & Frankham, 2004). Further, pathogen dynamics can be inferred directly using pathogen genetic data (Archie, Luikart, & Ezenwa, 2009; DeCandia, Dobson, & vonHoldt, 2018) and incorporated into landscape genetic analyses. Understanding specifically how infectious agents respond to the influence of landscape factors on hosts enables us to predict how such agents might spread based on present landscape configurations, as well as under potential future landscape scenarios (Real & Biek, 2007). This knowledge can subsequently inform management efforts at the population level (such as vaccination targeted at key regions, culling), as well as broader decisions relating to the management of the landscape itself, which is a key aim of landscape genetics generally (Manel & Holderegger, 2013; Segelbacher et al., 2010). Landscape genetics is being applied by managers at relatively low rates compared to related ecological fields such as landscape ecology, conservation biology and telemetry research (Bowman et al., 2016). Therefore, studies that contribute to the management of disease agents within populations could increase the practical impacts of landscape genetics significantly. However, the conceptual underpinnings of pathogen landscape genetics are not fully developed, and the methodologies employed are diverse and potentially confusing for new practitioners.

Here, we investigate how landscape genetic techniques are being used to better understand dynamics of microorganisms infecting host species. In conducting this review, we aim to both advocate and facilitate landscape genetic research involving disease‐causing organisms. We first evaluate the use of landscape genetics in disease ecology, including the types of questions addressed, the approaches used and the infectious agents studied. We then review established landscape genetic methods and their realized and potential applications to disease ecology. At last, we identify emerging frontiers in the landscape genetic study of pathogens that hold significant potential for advancing research in this field.

Landscape genetics was first implemented in the study of rabies virus by Real et al. (2005), offering an approach to overcome many feasibility issues associated with understanding landscape influences on pathogen transmission. The landscape genetic approach to studying disease was later reviewed by Biek and Real (2010), who were optimistic about its growth and future use. In particular, they noted that microparasites, such as viruses, are well‐suited to landscape genetic study due to their rapid mutation rate and potential spatial genetic structure that can be compared to heterogeneous landscape features at fine temporal and spatial scales. Analyses could be conducted using both pathogenic organisms and agents that do not cause significant diseases in their hosts (Biek, Drummond, & Poss, 2006). They also identified that methodologies such as GIS, which are commonly employed both in the wider landscape genetics literature and in spatial studies of infectious disease, had not been widely implemented in molecular epidemiology (Archie et al., 2009). Further, other popular landscape genetic tools, such as those focused on differential landscape permeability (e.g., least‐cost paths), were greatly underused despite compatibility with pathogen spatial genetic data.

Similar to landscape genetics, landscape epidemiology is an interdisciplinary field undergoing rapid development driven by technological advancements, and arguably still working to develop clear directions for future research (Meentemeyer et al., 2012). It is therefore likely that the interface of these two fields (i.e., where landscape genetics is used in epidemiology) is similarly challenged, perhaps to the extent that its potential is remaining unrealized. We thus believe it is timely to revisit the body of research that combines landscape genetics and landscape epidemiology, leveraging the work done both prior and subsequent to Biek and Real's (2010) earlier review into clear directions for future research.

## 2 CURRENT APPLICATIONS OF LANDSCAPE GENETICS IN DISEASE ECOLOGY

### 2.1 Literature search

We conducted a literature search in February 2018 using the ISI Web of Science database with the following terms:

TS=((“landscape genetic*” OR “landscape genom*”) AND (disease* OR pathogen* OR parasit* OR virus* OR virol* OR epidem* OR infect* OR transmi*))

The search returned 133 results. We read each article and retained the 51 empirical papers that used landscape genetic methods to address questions related to pathogens (see Supporting Information Appendix S1). We excluded reviews (*n* = 15), meeting abstracts (*n* = 1), purely methods‐based papers (*n* = 6) and articles that identified as or mentioned landscape genetics but did not sufficiently incorporate landscape factors or genetic data into the study (*n* = 32), studies that referred to any of our pathogen‐related search terms without it being a primary motivation for the study (*n* = 21), and studies that used words like “transmit” or “parasite” outside of the context of infectious agents (such as the transmission of behaviours) (*n* = 6). One paper was excluded due to a lack of access at our institutions. Studies that qualitatively discussed landscape with respect to genetic variation were kept, although one might argue that landscape genetics requires quantitative testing of landscape effects. We classified each paper according to the type of host system studied (plant, wild animal, domestic animal and human), the type of pathogen studied (bacterium, protozoan, virus, prion, fungus, macroparasite and transmissible cancer) and the source of genetic data (host, pathogen and vector), and we estimated the severity of disease that each studied pathogen causes in its sampled host or vector. We also categorized each article according to its general conceptual approach. Most examples described in this study were found in our literature search, while several other examples were cited by papers from our search and subsequently also discussed here.

**Figure 1:**
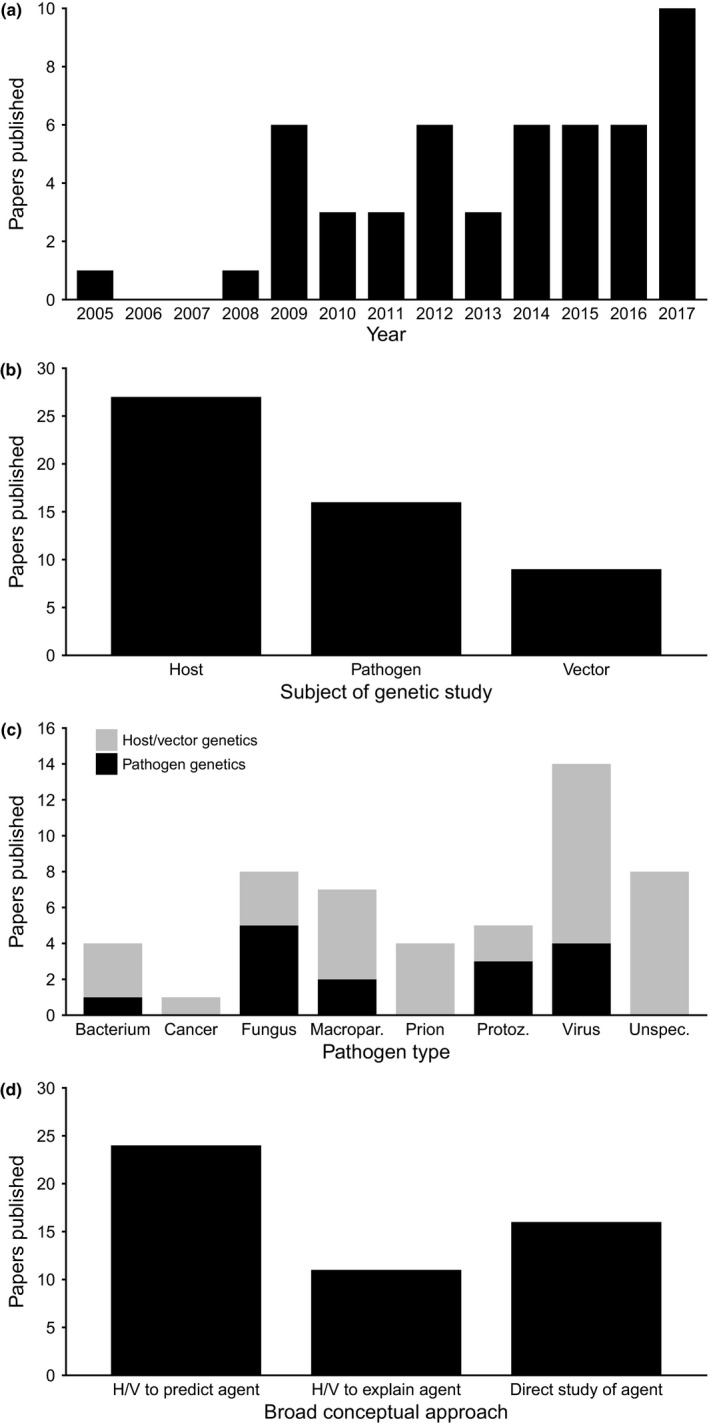
Papers using landscape genetic approaches for the study of infectious agents. (a) Number of publications per year that met our search criteria. (b) Number of publications using genetic data from each of the host, agent or vector species. (c) Number of publications studying pathogens by type, with genetic data source indicated for each type (“unspecified” typically involves studies of a hypothetical agent or estimates of overall pathogen exposure, such as inferred by immune‐linked loci). (d) Number of publications adopting each of our broadly identified conceptual approaches for applying landscape genetics to the study of pathogens/infectious agents—using host/vector genetics to predict agent spread, using host/vector genetics to explain agent spread/distribution and using pathogen genetics to directly study agent spread

Following publication of the first study using landscape genetics to investigate disease in 2005, there was little further research in this area until 2009, which saw a rapid increase in the number of publications (Figure 1a). This increase coincided with two prominent review articles (Archie et al., 2009; Biek & Real, 2010) that were strong proponents of a landscape genetics approach to disease ecology and expressed optimism about its future use. The rate of publication has remained relatively steady (and arguably low) since then, with none of the subsequent 7 years recording more publications than in 2009, when six papers were published. However, 10 articles using landscape genetics to investigate disease were published in 2017, potentially indicating increasing interest in this area of research.

A majority of studies (27 of 51) used genetic data from the host for comparison with landscape features (Figure 1b). This is likely because DNA is easier to obtain from larger, free‐living hosts than for pathogens, and methods for genotyping and characterizing host spatial genetic variation are more familiar to landscape geneticists, who predominantly study free‐living organisms (Storfer, Murphy, Spear, Holderegger, & Waits, 2010). Among pathogens that are associated with a particular animal vector, the vector is often genotyped (9 of 14 studies of vector‐borne diseases), as vectors such as ticks or mosquitos are also easily sampled, and vector gene flow can be used as a proxy for pathogen spread. Vectors can be targeted for population control as a means of limiting pathogen spread, which makes their study of immediate relevance to wildlife and livestock managers (Townson et al., 2005). Pathogen genetic data are used in only 16 of 51 pathogen landscape genetic studies, which was somewhat surprising considering that the pathogen is the primary motivation behind many of the reviewed studies. One study included both host and pathogen genetic data (Talbot, Vonhof, Broders, Fenton, & Keyghobadi, 2017).

Viruses were the most frequently studied type of infectious agent (14 of 51 studies; Figure 1c). In general, viruses evolve more rapidly than other microparasites, which makes them well‐suited to study of genetic variation for inference of transmission history (Archie et al., 2009; Grenfell et al., 2004). However, a majority of landscape genetic studies involving viruses used host genetic data, potentially reflecting the relative difficulty of obtaining viral data, which we discuss later in this section. Instead, the high representation of viruses is largely due to the considerable effort devoted to studying rabies, which comprised half of all landscape genetic studies on viral systems. Rabies is one of the most well‐known wildlife pathogens globally, due to its negative impacts on wildlife, domestic animal and human health (Gordon et al., 2004). Large outbreaks have occurred in North American and European wildlife in recent years, where considerable resources have been devoted to its management (Holmala & Kauhala, 2006; Slate et al., 2009). Animals infected with rabies also often exhibit behavioural changes that may make them easier to identify (Lefèvre et al., 2009), potentially aiding sampling of infected individuals.

We broadly define three distinct conceptual approaches by which landscape genetics has been used to study infectious agents (Figure 1d). These are the prediction of agent spread using genetic information from the host or vector; the use of host or vector genetic information to explain existing spatial variation in infection risk or prevalence; and the use of genetic information from the infectious agent to directly study transmission and spread. The remainder of this section will address each of these approaches in turn.

### 2.2 Host or vector genetic variation as a predictor of agent spread with respect to landscape

Because the spread of many microparasites (particularly directly transmitted forms) is facilitated by movement of free‐living hosts or vectors, the risk of spread of the agent with respect to heterogeneous landscape features can be estimated by relating those features to host/vector gene flow. This approach represents a direct application of the conventional landscape genetic paradigm to the study of disease transmission, where a typical animal landscape genetic study is interpreted in the context of the pathogenic organism. This can provide useful indications of the potential for individuals carrying pathogens to disperse across particular landscape features, which can be used to inform management efforts. For example, DeYoung et al. (2009) identified long‐distance gene flow among grey fox populations in Texas that was unrelated to landscape features tested, determining that current rabies oral vaccination plans should be expanded given the high potential for long‐distance host movement. In another rabies study, landscape genetics was used to characterize striped skunk dispersal across riverine and highway barriers to assess their utility as barriers to pathogen spread (Talbot, Garant, Paquette, Mainguy, & Pelletier, 2012).

Using host or vector genetic data to predict pathogen spread is attractive as it avoids sampling of the agent itself, which may be substantially more difficult, especially in wildlife populations. Identification of infected hosts often requires laboratory testing and may require specific, potentially invasive sampling approaches (e.g., necropsy) for accurate diagnosis. In addition, extensive sampling may be required to obtain adequate sample sizes when prevalence is low and must be conducted strategically to capture spatial heterogeneity. Direct study of pathogens may not be possible when predicting the risk of spread in as‐yet uninfected populations, or where identification of infected individuals is unreliable. However, a direct association of host or vector gene flow with spread of the microparasite should not be assumed given the potential influence of other factors such as other host and/or vector species, environmental persistence, pathogen reproductive mode or simply transmission via movement of nonreproducing hosts (Mazé‐Guilmo, Blanchet, Mccoy, & Loot, 2016; Tesson et al., 2016). For example, Lee et al. (2012) showed disassociation between host and virus genetic structure owing to host movement events that did not result in host gene flow (reproduction), but did result in transmission of feline immunodeficiency virus in bobcats. Indeed, correlation between host dispersal and parasite genetic structure is often weak (Mazé‐Guilmo et al., 2016). Therefore, studies using host or vector data alone have limitations for inferring or predicting pathogen spread, or lack thereof, directly. However, host landscape genetic studies can provide indications of the potential risk of spread of infectious agents, and the understanding gained about host movements can inform subsequent studies of pathogen dynamics.

### 2.3 Relating spatial heterogeneity in infection risk with host spatial genetic variation

Spatial variation in pathogen prevalence or infection risk can be represented in much the same way as any landscape variable (Escobar et al., 2017), making spatial data relating to presence of an infectious agent well‐suited for incorporation into host landscape genetic models. While spatial heterogeneity in pathogen prevalence could also be considered a component of the landscape that may influence spatial genetic variation in the host, typically only adaptive loci are investigated in this context. More commonly, host neutral genetic variation is used to explain spatial patterns of infection risk or prevalence. A prominent example is a study of chronic wasting disease (CWD) in white‐tailed deer. Blanchong et al. (2008) found that populations with lower CWD prevalence showed higher genetic differentiation from those that had high CWD prevalence. This genetic differentiation was found to be associated with roads and rivers, which were likely barriers to both host gene flow and CWD spread. These inferences have subsequently informed and been verified by additional landscape epidemiological research (Robinson, Samuel, Rolley, & Shelton, 2013).

Spatial heterogeneity in pathogen infection risk can also drive microevolutionary responses in the host (Epstein et al., 2016; Monello et al., 2017). Host species are constantly being challenged by parasitic organisms, which, if not overcome, cause disease and can have fitness consequences. This can create strong selection that acts on various genes, and geographic variation in selection at loci that are known to be associated with adaptive immune genes may reflect variation in pathogen pressure, and individual infection or disease risk (Fumagalli et al., 2011). This variation may be tested for association with environmental features such as temperature, humidity or urbanization (Tonteri, Vasemägi, Lumme, & Primmer, 2010), enabling insights into how future changes in climate or land use might influence overall pathogen prevalence.

### 2.4 Pathogen genetic variation to quantify pathogen transmission and spread

Using the sampled disease agent as the source of genetic data is the most direct way to infer pathogen spread across landscapes, but can be challenging to accomplish. Genetic material may be absent from, or uninformative in some infectious agents, such as prions or clonally transmissible cancers, necessitating genetic analysis of the host (Kelly et al., 2014; Storfer et al., 2017). In addition to the aforementioned difficulties with pathogen diagnosis, pathogen nucleic acid can be difficult to isolate from samples taken from the host or vector and would ideally be present in the blood, saliva or other easily collected sample. Samples may also require enrichment to obtain sufficient quantities of genetic material for analysis, which can be difficult to accomplish for many pathogens, particularly viruses. However, genetic information from viruses may be particularly useful for molecular epidemiologic analyses due to their rapid mutation rate that can closely infer transmission history (Archie et al., 2009; Brunker, Hampson, Horton, & Biek, 2012). Further, viruses are prominent emerging pathogens and have relatively small genomes, aiding whole genome‐analysis. Landscape effects on viral transmission are typically studied using phylogenetic approaches (Fountain‐Jones, Craft et al., 2017; Joannon, Lavigne, Lecoq, & Desbiez, 2010; Streicker et al., 2016; Young et al., 2017). To date, pathogens with larger and more slowly mutating genomes, such as protozoans (Carrel et al., 2015; Lo et al., 2017) and fungi (Brar et al., 2015; Rieux, De Bellaire, Zapater, Ravigne, & Carlier, 2013), have been studied using population genetics‐based methods with highly variable microsatellite and SNP loci. New methods based on next‐generation sequencing technologies such as targeted enrichment techniques (Lee et al., 2017) are helping to address challenges with sequencing viruses and other pathogens, facilitating greater use of pathogen genetic data in future landscape genetic studies.

The pathogenicity of an infectious agent and the length of its period of infection are other factors that may determine its utility for landscape genetic study. Highly pathogenic agents (i.e., those that cause a greater severity of disease) are typically of utmost interest due to their potential implications for wildlife conservation, agricultural production and human health. Among the studies identified in our literature search, agents that form chronic infections and have moderate or high pathogenic effects on their sampled host/vector organisms were more frequently investigated (see Supporting Information Appendix S1). However, genetically inferring transmission histories of pathogens in host populations experiencing high rates of mortality may be problematic because hosts through which the pathogen has spread may no longer be present in the population and are unable to be sampled. Similarly, acutely infectious agents from which the host recovers after a short period of time may also evade sampling. These characteristics may be less problematic for agricultural populations where morbidity or mortality can be identified and samples collected immediately, but inevitably leave “breaks” in the inferred chain of transmission among wild populations. While complete sampling of wild populations is rarely possible in any case, obtaining adequate sample sizes is easier for apathogenic or low‐pathogenicity agents that form chronic infections and may be sampled at any time postinfection (e.g., feline immunodeficiency virus; Biek et al., 2006; Lee et al., 2012; Fountain‐Jones, Craft et al., 2017). Landscape genetic study of such “model” infectious agents may be used to target specific ecological questions and provide insights into how similarly transmitted agents with higher pathogenicity might spread in the event of an outbreak.

## 3 COMMON METHODOLOGICAL APPROACHES IN LANDSCAPE GENETICS AND THEIR USE IN STUDYING PATHOGEN DYNAMICS

There are a variety of methods available for implementing landscape genetics, some designed specifically for landscape genetics, while others have been adapted from other fields. The rapid development of landscape genetics means that new methods are regularly emerging, and it is difficult to comprehensively review all of them. However, there are some well‐established methodological approaches that have either seen wide use for some time or are becoming increasingly popular at the cutting edge of the field (Balkenhol, Cushman, Storfer, & Waits, 2016). We describe the approaches (Table 1) and discuss their implementation in the study of pathogen transmission and spread.

**Table 1:** Common landscape genetic approaches and their potential use in pathogen research

Landscape genetic approach	Their potential applications in pathogen research	Pathogen landscape genetic examples
Landscape genetic simulation modelling	Predict pathogen spread in future landscape scenarios; predict spread of genes relevant to host–pathogen–vector interactions; test and validate new methods	Rees et al. (2008), Landguth et al. (2016), Leo et al. (2016)
Clustering and assignment methods	Detect barriers to pathogen spread and infer levels of barrier permeability; detect pathogen or vector environmental niche variation	Cullingham et al. (2009), Cote et al. (2012), Addis et al. (2015), Brar et al. (2015)
Landscape resistance surfaces	Identify probable transmission routes or corridors; identify hosts and vectors responsible for pathogen spread; predict effects of environmental change on pathogen spread	Liang et al. (2014), Streicker et al. (2016), Lo et al. (2017), Young et al. (2017)
Graph theory and network models	Genetic inference of host contacts; identify key habitat patches/populations contributing to pathogen spread	None
Genomic approaches	Identify associations of known candidate loci with spatial variation in pathogen exposure; infer spatial variation in pathogen exposure in different landscapes using associated loci; identify alleles determining disease susceptibility and incorporate the distribution of these into predictions of future pathogen spread	Garroway et al. (2013), Larson et al. (2014), Roffler et al. (2016), Wenzel et al. (2016)

### 3.1 Simulation modelling to test theoretical and predicted scenarios and validate methodology

In landscape genetics, simulation models are usually agent‐based and spatially explicit (Landguth, Cushman, & Balkenhol, 2016). Genetic data are modelled for individuals which have discrete spatial locations with respect to one another and with respect to environmental heterogeneity. Individuals move, behave and reproduce according to their own attributes in response to other individuals and in response to the simulated environment, and the model simulates changes in allele frequencies in response to these parameters. Landscape genetic simulation modelling has been used to test and validate methodological approaches (Cushman, Wasserman, Landguth, & Shirk, 2013; Zeller et al., 2016), address theoretical questions about how and why landscape heterogeneity influences genetics (Landguth et al., 2010), and evaluate and explain empirical observations (Shirk, Cushman, & Landguth, 2012). Further, simulation modelling can predict how a system might respond to certain changes, such as habitat fragmentation or future management activities.

Simulation modelling has been widely implemented in the study of pathogenic and nonpathogenic disease, beginning with medical research in the 1960s (Elveback & Varma, 1965). Frequently, epidemiological simulations are used to predict the spread of pathogens and their effect on host populations (Calonnec, Cartolaro, Naulin, Bailey, & Langlais, 2008). However, the use of landscape genetic simulations in pathogen studies has been relatively limited. Landscape genetic simulations have been used to predict raccoon rabies transmission risk across a river barrier by simulating various rates of host dispersal and comparing these outputs with empirical genetic data from the host (Rees et al., 2008). The spread of particular host genes relevant to disease can also be simulated to inform management efforts. For instance, Landguth, Holden, Mahalovich, and Cushman (2017) used landscape genetic simulations to determine optimal planting regimes to maximize the spread of blister rust resistant genes among whitebark pine populations. Such simulations could undoubtedly be applied to vector species in particular, such as predicting the spread of pesticide resistance genes in mosquitos (Chang et al., 2016) and selecting appropriate sites for introduction of genetically modified vectors (Lavery, Harrington, & Scott, 2008). In addition, with the need to develop further landscape genetic frameworks for the study of pathogens, simulation modelling can prove useful in testing and validating these techniques, as it has done in the broader landscape genetics field (Cushman et al., 2013; Zeller et al., 2016). For example, Leo, Gonzalez, Millien, and Cristescu (2016) used landscape genetic simulations to validate their multitaxa integrated landscape genetic framework, which appears to be a promising solution to the challenge of studying pathogens with multiple hosts and/or vectors. Landscape genetic simulations may also include epidemiological parameters such as mortality or activity responses to infection, or limited infectious periods, which may otherwise confound conventional (i.e., nonsimulation) landscape genetic approaches.

### 3.2 Clustering and assignment methods for quantifying connectivity and identifying transmission origin

Landscape genetic clustering and assignment methods have largely built upon classical methods from population genetics (e.g., principal components analysis, STRUCTURE, Pritchard, Stephens, & Donnelly, 2000) by incorporating spatial information (e.g., GENELAND, Guillot, Mortier, & Estoup, 2005; sPCA, Jombart, Devillard, Dufour, & Pontier, 2008) and environmental heterogeneity (e.g., constrained ordination, Anderson & Willis, 2003; POPS, Jay, 2011) into estimates of population structure and providing quantitative estimates of ancestry for each individual (François & Waits, 2016). Clustering methods have been relatively popular in studying pathogens and implemented for the inference of landscape barriers affecting both host (Addis, Lowe, Hossack, & Allendorf, 2015; Cote, Garant, Robert, Mainguy, & Pelletier, 2012; Cullingham, Kyle, Pond, Rees, & White, 2009; Frantz, Cellina, Krier, Schley, & Burke, 2009) and microparasite (Brar et al., 2015; Rieux et al., 2011) spatial genetic variation. Edge detection methods, such as Monmonier's maximum difference algorithm, (Monmonier, 1973) have also been used to detect landscape barriers to transmission in pathogen studies (Carrel et al., 2015; Joannon et al., 2010). Ancestry estimates from model‐based clustering algorithms can assign individuals to their populations of origin, enabling inference of landscape barrier permeability through the identification of migrants and thus estimation of the risk of pathogen spread across the barrier.

Most of the studies implementing clustering and assignment methods did not use approaches that incorporate environmental data. Instead, spatially or nonspatially explicit methods were typically used to identify genetic discontinuities and relationships with landscape barriers were inferred ad hoc, or analyses proceeded to entirely different methods that explicitly include environmental data. Associations between genetic discontinuities and landscape barriers should be considered with care due to the potential effect of intrinsic isolation mechanisms on genetic structure. For instance, studies on apple scab identified two distantly related lineages that are reproductively isolated through host specificity, but which have formed a narrow secondary contact zone in orchards where multiple host species are cultivated (Lemaire et al., 2016; Leroy, Lemaire, Dunemann, & Le Cam, 2013). Secondary contact zones occurring at equivalent spatial scales to that of landscape heterogeneity may result in genetic discontinuities resembling a barrier effect and thus be misattributed as such.

Other applications of clustering methods that explicitly integrate landscape variables, such as detecting environmental niche variation (Pease et al., 2009) and ancestry–environment relationships (Jay et al., 2012), remain relatively unexplored among studies of pathogen dynamics. These applications could translate in infectious organisms to the identification of distinct ecotypes or identify landscape features that coincide with infection foci or sources of pathogen spread. However, it must be noted that many of the genetic clustering and assignment methods presented here rely on classical population genetics models that generally do not apply to microorganisms. Therefore, the use of such methods in the study of pathogens is often limited to the inference of pathogen movement using host gene flow, with the exception of some fungal pathogens (Brar et al., 2015; Rieux et al., 2011). Some recent methods for identifying spatial population structure are free of classical population genetic assumptions, such as LOCALDIFF (Duforet‐Frebourg & Blum, 2014), EEMS (Petkova, Novembre, & Stephens, 2016) and MAPI (Piry et al., 2016), and can be applied to pathogens directly without these potential constraints.

### 3.3 Resistance surface modelling can identify transmission pathways and quantify spread by hosts and vectors

Resistance surfaces are commonly used in landscape genetics for modelling hypotheses concerning the influence of landscape features (from GIS landscape variables) on functional connectivity using techniques such as least‐cost paths (Adriaensen et al., 2003) or circuit theory (McRae, Dickson, Keitt, & Shah, 2008). These techniques produce measures of landscape or “effective” distance among populations or individuals for each hypothesis, which can be tested against observed genetic variation. The primary applications of resistance surface modelling in landscape genetics have been the identification of dispersal corridors and predicting the impacts of landscape and environmental change, such as habitat fragmentation or climate change, on connectivity. Similar to that, landscape genetic resistance surfaces can identify transmission corridors or future patterns of spread (e.g., Streicker et al., 2016), and such tools have been identified previously as having great utility for pathogen landscape genetic studies (Biek & Real, 2010). However, resistance surface modelling remains infrequently applied among pathogen studies. Careful consideration is required for identifying the most relevant landscape variables to be tested and correctly parameterizing (assigning costs to) the resistance surface(s) so that these variables are represented in a biologically meaningful way. Developing landscape resistance hypotheses for transmitted agents may be more difficult as their interactions with the landscape are often indirect, mediated by the ecology of hosts and vectors. Pathogen ecological niche models offer an empirical approach for constructing resistance surfaces based on ecological factors influencing pathogen prevalence (Escobar et al., 2017; Fountain‐Jones, Pearse et al., 2017), but these also may not adequately represent host/vector movements.

Our literature search returned only one study that explicitly modelled landscape resistance based on pathogen‐specific biology, testing elevation (as a proxy for temperature) as a predictor of *Plasmodium* spread, in addition to resistance surfaces that modelled human movements and mosquito vector ecology (Lo et al., 2017). However, several other studies applied resistance surfaces to hosts and vectors. Young et al. (2017) tested resistance surfaces based on waterbird niche models against genetic data from avian influenza. Two further examples of resistance surface modelling used host genetic data: Liang, Liu, Liao, and Gong (2014) studied landscape resistance of the snail *Oncomelania hupensis* to infer transmission of its parasite *Schistosoma japonicum*; while Rioux Paquette, Talbot, Garant, Mainguy, and Pelletier (2014) identified likely dispersal corridors for two rabies hosts. Further, Streicker et al. (2016) used resistance surfaces to construct least‐cost pathways predicting future spread of vampire bat rabies, and Barton, Gregory, Davis, Hanlon, and Wisely (2010) tested landscape resistance to rabies gene flow among striped skunks using landscape variables believed important for host dispersal, as well as landscape variables found to be relevant to other rabies hosts. Resistance surface modelling has thus demonstrated utility in identifying landscape drivers of functional connectivity that can shape pathogen spread and should see increasing use as frameworks for the integration of host, vector and pathogen data continue to develop.

### 3.4 Graph theory and network models—integrating landscape genetic and epidemiological approaches

Graph theoretical approaches, which describe connections (edges) between discrete objects (nodes) (Newman, 2003), are a flexible yet powerful tool for use in landscape genetics (Dyer, Nason, & Garrick, 2010; Garroway, Bowman, Carr, & Wilson, 2008). In landscape genetics, nodes can represent individuals, populations or habitat patches, possessing genetic parameters such as diversity measures (Dyer et al., 2010), or landscape parameters such as percentage habitat or habitat quality (Murphy, Dezzani, Pilliod, & Storfer, 2010). Similar to that, edges can represent genetic relationships between nodes such as genetic distances, gene flow or dispersal (Decout, Manel, Miaud, & Luque, 2012), or spatial/landscape relationships such as geographic distance or landscape resistance (Dyer et al., 2010). Distinct from other landscape genetic analytical approaches, graphs allow inferences based on the overall shape, or topology, of the network, which can provide unique insights into systemwide processes, such as hierarchical population structure (Dyer & Nason, 2004).

Network topology may be used to identify populations or habitat patches that form important “stepping stones” for maintaining genetic connectivity across an entire system. Such an approach enables experimental simulation whereby nodes may be selectively removed and the overall effect on the system's topology (e.g., overall connectivity, population structure) assessed. Metrics pertaining to the importance of individual nodes to network topology can be correlated with variables such as landscape to identify important drivers of network processes. Despite their unique applications, graph theory and network approaches are relatively underutilized in landscape genetics compared to methods specifically derived from population genetics and landscape ecology. However, among studies of infectious agents, network approaches in wildlife are becoming increasingly popular (Craft, 2015; Craft & Caillaud, 2011). Epidemiological network models are typically based on host contact networks, which are usually constructed using direct observations or indirect techniques such as mark‐recapture, telemetry or proximity loggers, and pathogens are simulated on these contact networks. Such approaches have already incorporated landscape and other environmental features. In addition, the potential for inferring host contacts in network models using pathogen genetic markers (see below) has been acknowledged in recent reviews (Craft, 2015; Gilbertson, Fountain‐Jones, & Craft, 2018; White, Forester, & Craft, 2017), and some studies have directly compared host contact network parameters to parasite genotypes (Bull, Godfrey, & Gordon, 2012). Despite this, to our knowledge, no published studies have used network models to investigate pathogen movement within a landscape genetic framework.

### 3.5 Genomic approaches to study microevolutionary responses to pathogens and landscape structure

While landscape genetics initially was used to investigate spatial genetic patterns using relatively few neutral markers, the more modern advent of landscape genomics allows the study of variation across the entire genome and effectively expands the scope of landscape genetics to include the study of functional, adaptive genetic variation. Next‐generation sequencing (NGS) techniques such as restriction‐site‐associated DNA sequencing (RADseq) require minimal prior knowledge of the genome under study and can genotype thousands of SNPs randomly distributed across the genome. Some of these SNPs will by chance be located within or near (and thus linked to) genes or regulatory regions that are under selection. Genomewide association studies (GWAS) can make use of this information to identify loci linked to phenotypic variation such as disease susceptibility. Genotyping of candidate loci identified using quantitative trait locus mapping and GWAS can be expanded across a large number of individuals using methods such as targeted sequence capture (Grover, Salmon, & Wendel, 2012), and these data can be tested in a landscape genomic framework for associations with environmental variables.

Loci exhibiting a signature of selection can be identified using outlier tests (Excoffier, Hofer, & Foll, 2009; Luu, Bazin, & Blum, 2017), which search for loci with allelic frequencies that are outliers relative to the majority. Such loci are considered potentially under selection and may then be tested *a posteriori* for correlations with environmental variables. Newer methods have focused on explicitly incorporating environmental variables into landscape genomic analyses, known as genetic–environment association (GEA) tests (Lotterhos & Whitlock, 2015; Rellstab, Gugerli, Eckert, Hancock, & Holderegger, 2015). GEA analyses test for correlations between environmental variables and individual genotypes, which eliminates problems due to underlying population structure that must be controlled when using outlier tests. NGS approaches also generate thousands of neutral loci, which provide greater power to detect fine‐scale neutral genetic structure than conventional studies based on relatively few loci (Allendorf, Hohenlohe, & Luikart, 2010). However, for studies with a particular focus on functional genetic variation, NGS approaches can also be adapted specifically for this purpose through targeted sequencing of the exome (Roffler et al., 2016) or transcriptome (de Wit & Palumbi, 2013).

While genomic technologies are becoming the norm in microbial research, approaches incorporating landscape (i.e., landscape genomics) have yet to see widespread usage. Current examples focus on established candidate loci from the host known to be relevant to immune function from prior research, such as in commercially important salmon (Larson, Seeb, Dann, Schindler, & Seeb, 2014; Tonteri et al., 2010). Wenzel, Douglas, James, Redpath, and Piertney (2016) used SNPs previously identified to be associated with nematode burden in red grouse (*Lagopus lagopus scotica*) using GWAS, as well as *F*_st_ outlier loci, to investigate parasite‐driven genetic structure across a landscape. Garroway et al. (2013) used SNPs identified from transcriptome sequencing of great tits (*Parus major*) to conduct a GEA with respect to avian malaria infection risk and contrasted this with neutral gene flow. Another targeted approach employed exon capture to genotype SNPs that were then subjected to outlier and environmental association tests to investigate variation in pathogen exposure with respect to environment (Roffler et al., 2016). The spread of functional alleles has also been incorporated into landscape genetic simulations (Landguth et al., 2017), enhancing predictions of future pathogen spread and its effects on host populations. This small body of research is promising for expansion of landscape genomic studies designed to couple pathogen‐related functional genetic variation with landscape variables.

## 4 EMERGING CONCEPTS FOR THE LANDSCAPE GENETICS OF INFECTIOUS AGENTS

While we believe that there remains much unexplored utility in established landscape genetic methods for the study of pathogen dynamics as we have described above, we also note new frontiers with significant potential for expanding research in this area. We complete this review by discussing three particularly promising frontiers.

### 4.1 Simultaneously integrating host, vector and landscape variables into studies of pathogen gene flow

**Figure 2:**
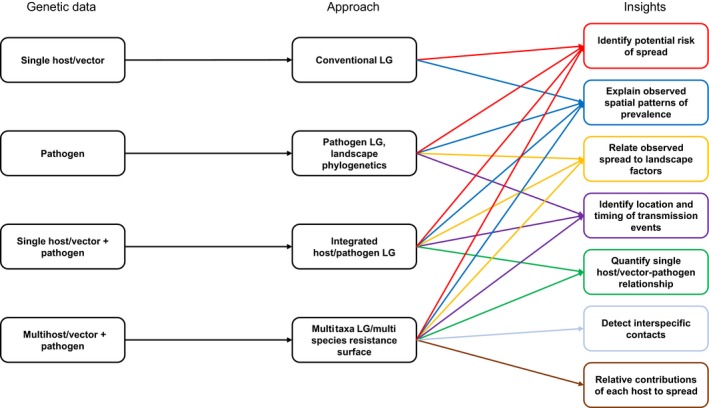
Schematic indicating the increasing insights that may be gained from using approaches that are able to integrate additional host/vector and pathogen genetic datasets, starting with genetic data from a single host or vector, through to multispecies approaches that integrate multiple host, vector and pathogen datasets. LG: landscape genetic

Studies relating pathogen genetic data directly to the landscape using resistance surfaces are challenged by the mediating influence of distinct host and vector traits, as well as relative differences in the contributions of multiple host and/or vector species to microparasite gene flow. This necessitates frameworks that more holistically incorporate multiple host and vector factors into studies of pathogen gene flow, which can expand the potential insights provided by landscape genetic studies of infectious agents (Figure 2). Single or multiple host or vector species can be added as “landscape variables” (e.g., as resistance surfaces) in addition to physical landscape and environmental variables to test as factors shaping spatial pathogen genetic structure. Resistance surfaces for tests of microparasite gene flow can represent host/vector distributions or abundance, ideally inferred from empirically derived ecological niche or species distribution models. Optimally, host/vector movement would be represented (Dougherty et al., 2018), using outputs from agent‐based movement models informed by telemetry or mark‐recapture data, or host/vector landscape genetic data representing spatial patterns of gene flow. We note that the common issue in conventional landscape genetics of spatio‐temporal mismatches between landscape processes and genetic change (Anderson et al., 2010; Landguth et al., 2010) would apply even more strongly here. Researchers must simultaneously consider the potentially different spatial and temporal scales over which host and pathogen genetic changes (and potentially those of additional host/vector species) and landscape changes occur. Nonetheless, with careful study design, such an approach has significant potential to unify hosts/vectors and landscape variables under a single analytical framework for explaining and predicting pathogen transmission and spread. Importantly, it allows a flexible framework for both single and multihost/vector systems.

Approaches integrating multiple host and vector datasets into landscape genetic studies of infectious agent gene flow have been proposed recently. Leo et al. (2016) developed a multitaxa integrated landscape genetic framework for diseases, which simultaneously quantifies the effects of both landscape variables and interspecific codispersal on pathogen gene flow in multi‐host‐vector systems. Few studies include both host and pathogen genetic data in landscape analyses. However, Fountain‐Jones, Craft et al. (2017) tested host relatedness, in addition to host demographic and landscape variables, and found this to be an important predictor of pathogen gene flow in a single‐host system of feline immunodeficiency virus in bobcats. A framework for combining multiple host and vector resistance surfaces was recently demonstrated for Chagas disease by Schwabl et al. (2017), involving subsequent validation using landscape genetic simulations. Although their approach does not explicitly include host and vector movement or gene flow, it advocates careful selection of landscape variables based on each host and vector species, informed by previous research. A recent study tested least‐cost path models of water bird movement estimated from ecological niche models, and road networks representing human movement, as potential predictors of avian influenza spread (Young et al., 2017). Other studies have investigated landscape genetic structure in multiple hosts of the same pathogens, identifying divergent dispersal patterns that could be integrated into studies of pathogen gene flow under such a framework (Rioux Paquette et al., 2014; Vander Wal et al., 2013). Approaches that consider whole ecological communities have recently been identified as necessary for advancing our understanding of pathogen dynamics (Fountain‐Jones, Pearse et al., 2017; Johnson, de Roode, & Fenton, 2015). Studies integrating multiple host and vector species into landscape genetic models of spread of infectious agents represent an important step towards such a paradigm.

### 4.2 Using molecular markers from infectious agents to detect cryptic landscape‐host processes

The rapid mutation of microparasites relative to their hosts has potential to provide greater power to detect subtle variation in host movement patterns in response to the landscape, as well as earlier detectability of changes in host movements (such as in response to a new barrier) that are yet to be reflected in host genetic structure (Landguth et al., 2010). In addition, movements of nonreproducing hosts are difficult to detect using host genetic markers, but instead might be inferred using markers from directly transmitted microorganisms. Such an approach has demonstrated the utility of a chronic, relatively apathogenic infection of felids (feline immunodeficiency virus) for identifying demographic structure of mountain lions and recent population history (Biek et al., 2006), and has identified movement of bobcats across a highway barrier that was not detectable using host markers (Lee et al., 2012). However, these approaches have not been broadly applied, particularly in the study of landscape effects.

The application of microparasite molecular markers to the study of host movements should be considered with some caveats in mind. As we have discussed previously, host movements and parasite transmission are not necessarily coupled (Mazé‐Guilmo et al., 2016), and assumptions about how closely parasite gene flow may reflect host movements must be made with care. Also, rapid mutation and rapid dissemination of new alleles (i.e., infectious agent transmission can generally occur much more quickly than host reproduction) largely limit the use of genetic data from the microparasite to the study of very recent or ongoing landscape changes, while older processes are better studied using host genetic data. However, choice of infectious agent based on its characteristics (e.g., mutation rate, prevalence in a population, mode of transmission) may be tailored to the type and age of the host process under study. We thus do not propose that microparasite genetic data alone should be used to study host movements in their entirety, but rather that it may have specific utility as a complementary approach to host markers for providing a more complete analysis of host movement. Such insights might include contacts between specific hosts, potentially including interspecific interactions. As new infectious agents are discovered and their relationships with host movements become better understood, microparasite molecular markers will have increasing utility in landscape genetic research of host populations.

### 4.3 The role of phylogenetics in understanding landscape influences on pathogen genetic variation

Phylogenetic approaches can reconstruct very recent epidemic histories, providing insights into particular transmission events and pathways that may be contextualized temporally and spatially (Corman et al., 2014; Faria et al., 2014; Carroll et al., 2015; Magee, Beard, Suchard, Lemey, & Scotch, 2015; Fountain‐Jones, Packer, et al., 2017; Fountain‐Jones, Pearse et al., 2017). The majority of such work has been conducted on RNA viruses owing to their small, rapidly mutating genomes, requiring relatively little sequencing effort to detect contemporary phylogenetic signals. Other pathogens that evolve more slowly, such as bacteria or fungal pathogens, require the sequencing of larger portions of their genomes to capture equivalent phylogenetic signals (Biek, Pybus, Lloyd‐Smith, & Didelot, 2015). While this is becoming increasingly feasible (Kao, Haydon, Lycett, & Murcia, 2014), more complex computational analysis is required to make meaningful conclusions.

Several approaches may be used for relating phylogenetic information with landscape variables. Neighbour joining trees can identify clusters for quantifying population‐level landscape genetic relationships (Joannon et al., 2010). The calculation of genetic distances based on maximum likelihood trees (Carrel, Emch, Tung, Jobe, & Wan, 2012; Real et al., 2005; Young et al., 2017) results in distance matrices that can be correlated with landscape resistance matrices using conventional landscape genetic approaches. Relaxed random walk phylogeographic approaches (Lemey, Rambaut, Welch, & Suchard, 2010) that can reconstruct pathogen dispersal have been linked to landscape predictors using a “phylogeographic GLM” method (Faria, Suchard, Rambaut, Streicker, & Lemey, 2013; Jacquot, Nomikou, Palmarini, Mertens, & Biek, 2017). The phylogeographic GLM approach has enabled a better understanding of how landscape and hosts can constrain pathogen spread. For example, using the phylogeographic GLM approach on viral genomic data, roads and rivers, coupled with dog distribution, were found to impact rabies spread in Tanzania (Brunker et al., 2018). However, this approach is limited to discrete sampling locations and is computationally intensive (Dellicour, Rose, & Pybus, 2016). A recent framework by Dellicour et al. (2016) modifies the phylogeographic GLM approach to use resistance surfaces to efficiently quantify landscape resistance along transmission pathways inferred by continuous phylogeographic analyses. These landscape resistances are then correlated with temporal estimates of transmission along these routes to estimate how the landscape has shaped rates and directions of pathogen spread. Such approaches are yet to be broadly applied, but appear to be important developments that should see increasing application in the future.

## 5 CONCLUSION

Overall, landscape genetics has been relatively underutilized in disease ecology research. We believe this is partly due to a lack of cross‐disciplinary awareness between the two fields, but also a lack of a clear landscape genetic framework specifically designed for tackling pathogen systems, which are often complex and do not facilitate easy translation of existing landscape genetic tools. However, we note there has been a recent effort to develop new frameworks for such research, expanding the utility of the landscape genetic toolset. These tools will increase our capacity to study complex multihost and host‐vector systems, improving the integration of multiple genetic datasets and accounting for interspecific interactions. Improved understanding of host–parasite associations will facilitate the use of microparasite genetic markers to provide insights into host processes that may be difficult to detect using conventional host landscape genetics. Identification of idealized systems that are designed to target specific ecological questions will also facilitate progress in this field. Recent methods that enable the incorporation of quantitative landscape data into spatio‐temporal phylogenetic reconstructions of recent transmission events, coupled with advances in high‐throughput sequencing, hold great promise for studying how the landscape shapes transmission processes. We believe that these recent developments represent a renewed interest in advancing landscape genetic research in pathogen systems, which we expect will translate to continued growth of research in this area.

## CONFLICT OF INTEREST

None declared.
